# Investigation on Long-Term Stability of Vermiculite Seals for Reversible Solid Oxide Cell

**DOI:** 10.3390/molecules28031462

**Published:** 2023-02-02

**Authors:** Ruizhu Li, Yue Lu, Yutian Yu, Xianzhi Ren, Feng Ding, Chengzhi Guan, Jianqiang Wang

**Affiliations:** 1 Key Laboratory of Interfacial Physics and Technology, Shanghai Institute of Applied Physics, Chinese Academy of Sciences, Shanghai 201800, China; 2 Zhejiang CPS Cathay Packing & Sealing Co., Ltd., Hangzhou 311255, China

**Keywords:** reversible solid oxide cell, vermiculite seals, thermal cycles, long term operation

## Abstract

A reversible solid oxide cell (RSOC) integrating solid oxide fuel (SOFC) and a solid oxide electrolysis cell (SOEC) usually utilizes compressive seals. In this work, the vermiculite seals of various thickness and compressive load during thermal cycles and long-term operation were investigated. The leakage rates of seals were gradually increased with increasing thickness and input gas pressure. The thinner seals had good sealing performance. The compressive load was carried out at thinner seals, the possible holes were squeezed, and finally the leakage rates were lower. With a fixed input gas pressure of 1 psi, 2 psi, and 3 psi, the leakage rates of 0.50 mm vermiculite remained at around 0.009 sccm/cm, 0.017 sccm/cm and 0.028 sccm/cm during twenty thermal cycles, while the leakage rates remained at around 0.011 sccm/cm for about 240 h. Simultaneously, elemental diffusions between seals and components were limited, implying good compatibility. Furthermore, the open circuit voltage (OCV) remained at around 1.04 V during 17 thermal cycles, which is close to Nernst potentials. The stack performance confirmed that the vermiculite seals can meet the structural support and sealing requirements. Therefore, the vermiculite shows good promise for application in stacks during thermal cycles and long-term operation.

## 1. Introduction

The increasing energy demand in modern society has given rise to excessive consumption of fossil fuels and uncontrolled emission of greenhouse gases, resulting in global warming and environmental balance destruction. The conversion of electrical into chemical energy by electrolyzing water is meaningful for energy storage of renewable energies [1,2,3]. Simultaneously, the produced hydrogen could be used for electricity generation. A reversible solid oxide cell (RSOC) integrates solid oxide fuel (SOFC) and a solid oxide electrolysis cell (SOEC), which can directly convert chemical to electricity without pollution by combining with heat and power (CHP) and can store electricity from windmills, solar cells and nuclear energy by electrolyzing H_2_O and CO_2_ [4,5,6]. RSOC could meet the diverse demands in multipurpose scenarios and had drawn more interest benefiting from its optimized thermal compatibility of components and reduced fabrication cost. Among various designs of RSOC stacks, planar design has contributed a greater concentration due to easier fabrication, improved performance, and relatively high-power density.

To increase overall output/input voltage, cells, interconnects and seals are precisely assembled into stacks [7,8]. The seals can prevent gas mixing in the anode and cathode to ensure the stable operation of the stack. The seals in the stack are required between air electrode and interconnect, hydrogen electrode and interconnect, cell stack and metal frame. Therefore, high-temperature hermetic seals are necessary in a RSOC stack. The seals must at least meet multiple requirements including good gas tightness, thermal stability, interfacial compatibility, high electrical insulation, and mechanical properties [9,10]. Low gas leakage rates and high thermal cycle stability are particularly significant. Furthermore, long-term stability of seals remains an issue. Lack of suitable seals has greatly hindered the development of planar RSOC stacks.

Broadly, the seals of stack can be classified into compliant seals, rigidly bond seals, and compressive seals. Compliant seals are deformable materials which could concatenate cells and interconnects [11,12,13]. Only noble metals including platinum and gold could be applied in stacks for avoiding corrosion. However, they could not properly wet adjacent components and their cost is unacceptable in commercialization. Silver, copper, and Ag-Cu-Ti brazing seals were investigated. The joint between metal brazing and adjacent components would seriously degrade in an oxidizing and reducing environment, finally leading to pores and cracks forming. Besides, the metal brazing seals would delaminate after a short time because of the coefficient of thermal expansion (CTE) mismatching between the oxide layer and interconnect. The Cr_2_O_3_ scale in gold-brazed and silver-brazed seals are problematic. Hydrogen diffusion from fuel may lead to ‘hydrogen embrittlement’ and degrade mechanical properties. Cracks and defects would initiate and propagate at the interface, causing leaking holes. Mostly seriously, metals and metal-brazing seals are electrically conductive, which means an insulating layer must be used in metals. 

Rigidly bond seals could form a tight junction between seals and adjacent components. So far, most sealing developments have focused on rigidly bond seals, which are mainly categorized into glass and glass-ceramic seals [14,15,16,17,18,19]. The glass could infiltrate into adjacent components and form strong bonding at the interface. The advantages of rigid seals were that the CTE of materials could be adapted to adjacent components by controlling the phase constituent. However, several physical and chemical properties should be simultaneously obtained in RSOC sealing, which is a challenge. The significant physical properties coinhere glass transition temperature (T_g_), glass softening temperature (T_s_), crystallization behavior, CTE and electrical resistivity. Chemical properties include but are not limited to resistance to chemical reactions with vapor gas and resultant glass vaporization and degradation in high temperatures (500~800 °C) and in oxidizing and wet reducing atmospheres. Sr-O borosilicate glass would form LaBO_3_ with 3–25 mol% La_2_O_3_. Sr-containing lanthanum borate form LaSi_2_O_7_ at 800~1000 °C. Moreover, the crystallization phase of BaO-CaO-borosilicate glass increases from 50% to 70% after 120 h in air at 750 °C. The crystallization phase would have a harmful effect on sealing performance, including mismatching CTE, leading to cracks, increased electrical resistivity, and gas leakage [10,20,21,22]. Additionally, the vaporization of glass constituents, such as B_2_O_3_ forming B(OH)_2_ and SiO_2_ forming Si(OH)_4_, can adversely affect the thermal and electrical properties. Furthermore, glass/glass–ceramic seals are disadvantageous for long-term operation and have a tendency to react with interconnect and electrode at high temperatures [23,24,25]. Moreover, destructive dismantling of stacks is necessary because of malfunctioning components. 

Compressive seals are deformable under an external load and have been developed to overcome disadvantages of glass seals [26,27,28]. Compressive seals were developed to overcome the disadvantages of glass and glass–ceramic materials. Thermal expansion matching is not required because the seals are not rigidly fixed with adjacent components. This allows the cells and interconnects to expand freely during thermal cycles and long-term operation. Therefore, stacks are easy to repair, and the replacement of components becomes possible. Mica, mica-based hybrid seals, Al_2_O_3_-based hybrid seals, ceramic fiber and vermiculite are popular compressive seals. Mica is composed of small, discrete mica flakes, ideally oriented with their cleavage planes parallel to adjacent components. Single-layer and multi-layer mica, including phlogopites (KMg_3_(AlSi_3_O_10_)(OH)_2_) and muscovite (KAl_3_(AlSi_3_O_10_)(OH)_2_), provide a tolerant mismatching CTE with adjacent components during thermal cycles at 800 °C [29,30,31]. Thus, residual stress would be less. Muscovite mica would lose its constitutional water exceeding 600 °C, while phlogopite mica loses its constitutional water above 950 °C. This behavior would cause a swelling of mica that may not affect the sealing properties. Moreover, mica that achieves good sealing performance requires high compressive loads exceeding 6 MPa. Furthermore, hybrid mica with a compliant glass interlayer was required for reducing leakage rates [32,33]. In glass-infiltrated mica, leakage rates are decreased. However, the volatilization of B_2_O_3_ is inevitable. The long term of glass material is a challenge. Therefore, developing non-glass compressive seals with low leakage rates are essential. Ceramic fiber compressive seals are an insulation material of alumina-silica composition that resists oxidation and is easy to fabricate and install. However, it is flexible, having an elastic recovery higher than 93.35%. It is mostly used at high temperatures for long times, and its volume resistivity at 850 °C is more than 10^6^ Ω·cm. However, its porosity is higher than 90%. In order to reduce leakage rates, glass infiltration is also required. Selection of a stable infiltration is significant. Apart from leakage rate reduction, the infiltration must be insulating and have chemical and physical stability in a RSOC operating environment. However, the stability of infiltration between adjacent and fiber seals is a challenge. The fumed silica has a high dispersion and has a chainlike particle morphology forming colloid silica, which could bond together via weak hydrogen bonds to form a three-dimensional network. The colloid silica with a size much larger than that of fumed silica filled in the voids of the fiber, which could effectively reduce the leak rates. However, the long-term stability of seals is difficult in the complex environment of RSOC stacks. In Al_2_O_3_-based hybrid seals and other ceramic-based seals, cracks and pores in materials and defects at the interface contribute high leakage rates. The thermal and chemical stabilities are also inferior. In reality, those seals could not meet sealing requirements and have some disadvantages. A load frame is required to apply a constant compressive load during operation, which would introduce a uniform distribution of load. To improve sealing performance, a high compressive load is required, and a suitable thick layer is needed.

Vermiculite is a phyllosilicate mineral with an ideal chemical formula (Mg^2+^, Fe^2+^, Fe^3+^)_3_[(SiAl)_4_O_10_]OH_2_·4H_2_O, whose structure is similar to that of mica. It is mostly used in the manufacture of bio-nanocomposites, environmentally friendly flame-retardant products and solid oxide electrochemical device sealing [34]. Vermiculite is easy to fabricate and install and could be used at high temperatures for long-term operation in reversible solid oxide cell. Generally, the seals in a reversible solid oxide cell stack should operate continuously for more 5000 h in mobile applications, for more than 40,000 h in stationary application, and endure thermal cycles without degradation [10,20,35]. Materials should be compatible with adjacent components to avoid pores and interactions. Additionally, the seals should withstand mechanical stresses, temperature gradients, and stack weight or external loads during operation and transportation. Fulfilling requirements is difficult at the same time. Considering planar stack design, analyzing the compressive properties of seals is important. Suitable compressive loads between seals and cells are required to achieve electrical contact between cells and interconnects as well as achieving sealing performance. Meanwhile, the compressive load of a stack is limited by the properties of components. The compressive sealing performance is well exhibited under constant compressive loads. And achieving good electrical contact are also required at least some of load in planar stacks. Loads on an interconnect are concentrated on a small area, mainly leading to mechanical failure. The seal of various thicknesses is also important during RSOC operation. In this paper, we focused on the effect of long-term operation, thermal cycles, compressive loads, and the thickness of vermiculite seals on sealing properties.

## 2. Results and Discussions

### 2.1. The Investigation of Leakage Rates

**Figure 1:**
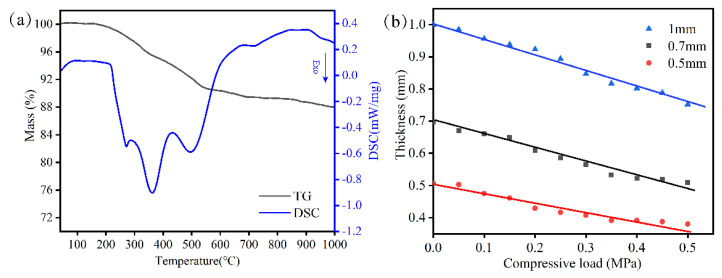
TG-DSC curves of vermiculite at a heating rate of 10 °C/min (**a**); function of thickness and compressive load (**b**).

The mass and thermal changes with temperature were measured to ensure a suitable heat treatment process. Figure 1a shows the mass decreasing percentage and thermal changes with temperature from 40 °C to 1000 °C in air. It was obvious that the weight loss of seals was drastic from 200 °C to 600 °C and then it had a little change above 600 °C. Combined with a combustion exothermic peak of DSC, the weight loss was from organic additives volatilizing. Accordingly, the heat treatment process was as follows. The samples were heated to 200 °C at a heating rate of 5 °C/min and stayed there for 1 h. They were then heated for 500 °C at a heating rate of 2 °C/min and stayed there for 1 h. Finally, the samples were heated to objecting temperature at a heat rate of 2 °C/min. The organic additives were slowly burned out from the samples without generating many holes. Accordingly, the relationship between thickness and compressive load is important in stack assembly. Figure 1b shows that the thickness is negatively correlated with a compressive load ranging from 0 to 0.5 Mpa. At a fixed compressive load of 0.3 MPa, the thicknesses of 0.50 mm, 0.70 mm and 1.00 mm were 0.40 mm, 0.57 mm and 0.87 mm, respectively. The predication of thickness under some compressive load is helpful for stack assembly. The coordination between assembly and compressive load could form achieve an electrical contact between cells and interconnects as well as achieve sealing performance.

**Figure 2:**
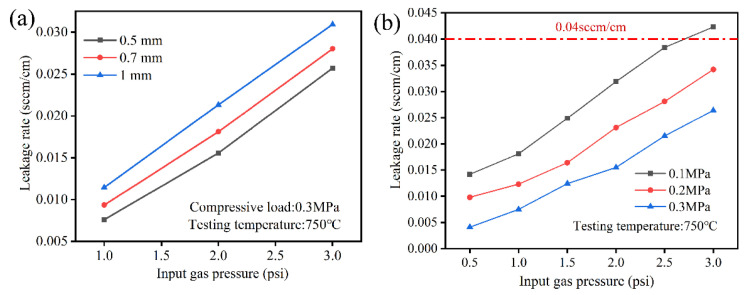
Effect of thickness (**a**) and compressive load (**b**) on leakage rates at 750 °C under input gas pressure of 1 psi to 3 psi.

In order to evaluate the effect of thickness and compressive load on sealing performance, the leakage rates were measured, as shown in Figure 2. It is obviously observed from Figure 2a that the leakage rates of vermiculite seals gradually increased with increasing thickness and input gas pressure. When the input gas pressure was fixed at 2 psi, the leakage rates were 0.015, 0.018, and 0.021 sccm/cm, corresponding to 0.50 mm, 0.70 mm, and 1.00 mm. The thinner seals had good sealing performance. Figure 2b shows the relationship between leakage rates and compressive load, exhibiting the decrease of leakage rates under increasing compressive load. Obviously, the leakage rates were slightly higher than 0.04 sccm/cm under an input gas pressure of 3 psi under a compressive load of 0.1 MPa, which exceeds the conventional requirements of seals. The higher the compressive load, the smaller the leakage rate under the same input gas pressure. The compressive load was carried out at seals, the possible holes were squeezed, and finally the leakage rates were lower. Further examinations were conducted to evaluate the application of various thickness seals.

**Figure 3:**
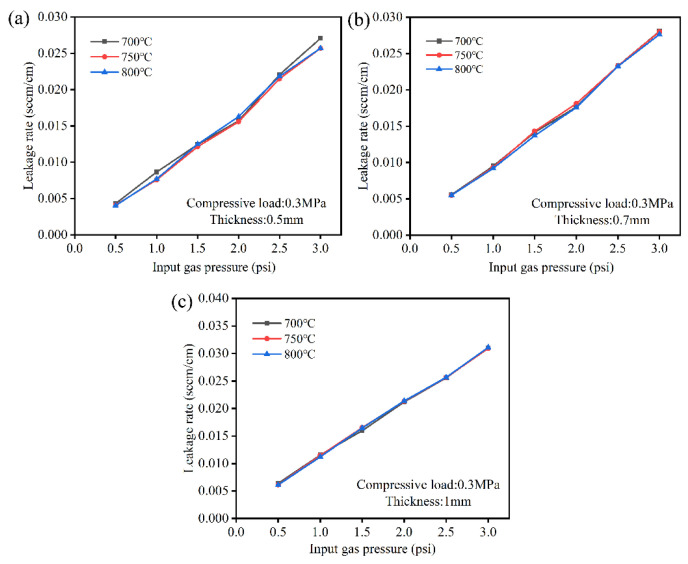
Effect of thickness and temperature corresponding to 0.5 mm (**a**), 0.7 mm (**b**), and 1 mm (**c**) on leakage rates under compressive load of 0.3 MPa.

Figure 3 shows that the effect of thickness on leakage rates under a compressive load of 0.3 MPa under input gas pressure ranging from 1 psi to 3 psi. All leakage rates were lower than 0.04 sccm/cm. The leakage rates increased with increasing thickness, where leakage rates were 0.009 sccm/cm, 0.010 sccm/cm, and 0.011 sccm/cm, corresponding to 0.1 MPa, 0.2 MPa, and 0.3 MPa at 750 °C under an input gas pressure of 1 psi. With increasing temperature, the leakage rates of 0.5 mm seals were similar under the same input gas pressure. At a fixed input gas pressure of 1 psi, the leakage rates were around 0.016 sccm/cm at temperatures ranging from 700 °C to 800 °C. The temperature had less influence on sealing performance, the thickness of seals is smaller, and the leakage rates are lower. Therefore, the thickness of seals should be considered during stack assembly.

**Figure 4:**
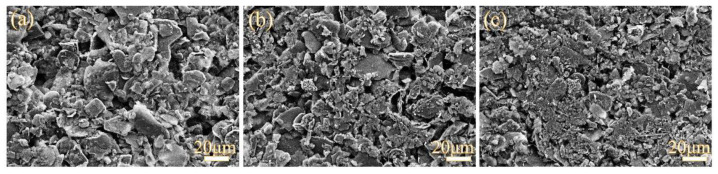
SEM micrograph of seals after being heated to 750 °C under 0.1 MPa (**a**), 0.2 MPa (**b**), and 0.3 MPa (**c**).

Furthermore, the profile after various tests maintained the original shape, depending on powder packing. Then, the surface micrographs of vermiculite seals were examined after heating to 750 °C under various compressive loads, shown in Figure 4. It was observed from the surface that the seals gradually became dense with increasing compressive load. The phyllosilicates were packed tightly in seals at high temperatures. Even though there were some holes, the leak channels were circuitous, and the leakage rates reduced with increasing compressive load from 0.1 MPa to 0.3 MPa. Combined with leakage rate results, it was indicated that thinner seals with a suitable compressive load exhibited good sealing performance, the phenomena being similar with leakage rates.

### 2.2. Evaluation of Long-Term Sealing Performance 

**Figure 5:**
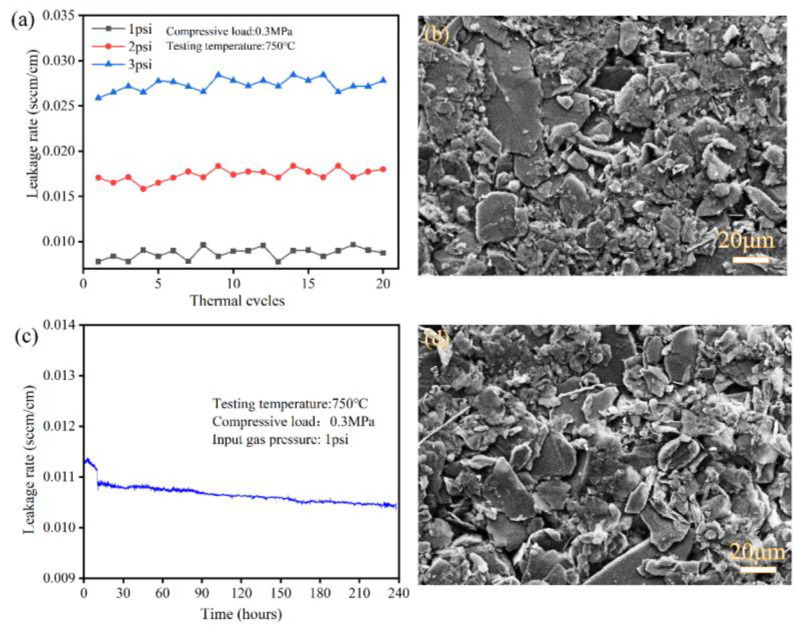
The leakage rates of vermiculite seals over 20 thermal cycles (**a**) and long-term operation (**b**) under a compressive load of 0.3 MPa and input gas pressure of 1 psi to 3 psi at 750 °C; and the SEM micrograph after 20 thermal cycles (**c**) and long-term operation (**d**).

To further examine long-term stability of vermiculite, the seals underwent long-term operation at 750 °C and twenty thermal cycles from 750 °C to 200 °C were conducted. Figure 5a,b show that the leakage rates of seals after each thermal cycle and long-term operation under a compressive load of 0.3 MPa with input gas pressure from 1 psi to 3 psi corresponding to SEM micrographs. The leakage rate remained stable at various gas pressures. For instance, with a fixed input gas pressure of 1 psi, 2 psi, and 3 psi, the leakage rates remained around 0.009 sccm/cm, 0.017 sccm/cm and 0.028 sccm/cm during twenty thermal cycles. The tendency of seals indicated that the micro-cracks remained stable and would not increase during thermal cycles. Figure 5c shows the variation of leakage rate over time at 750 °C under an input gas pressure of 1 psi. The leakage rate had a slight increase from 0.0112 sccm/cm to 0.0113 sccm/cm for three hours and then rapidly decreased to 0.0109 sccm/cm after 14 h. Finally, the rate exhibited a slight decrease, ranging from 0.0109 to 0.0105 sccm/cm. The phenomenon originated from the leak paths decreasing with extending heating time under a compressive load of 0.3 MPa. Combined with the SEM micrograph in Figure 5b,d, the phyllosilicates of the vermiculite seals were packed tightly over the thermal cycle and long-term operation and there were few new holes generated in seals, whose variation tendency was similar to leakage rates.

**Figure 6:**
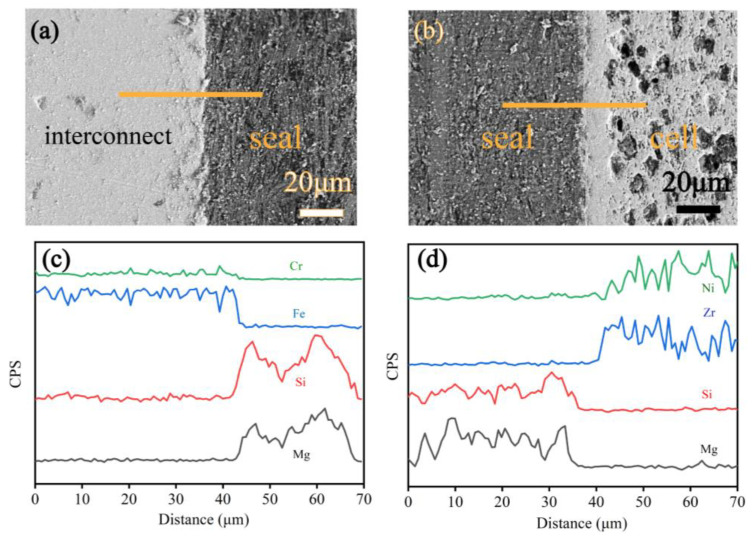
The SEM micrographs and EDS compositional profiles across the interface after 20 thermal cycles, under a compressive load of 0.3 MPa at 750 °C: seal/interconnect interface (**a**,**c**), seal/anode interface (**b**,**d**), exhibiting better interfacial compatibility.

The SEM micrograph attached with EDS line profiles of cross-sections were examined to analyze interfacial compatibility between seals and adjacent components. Figure 6a,b show the interfacial micrograph of interconnect/seal/cell after twenty thermal cycles. It indicates that contact at the interface remained complete and no obvious cracks were detected. Thermal cycle stability was achieved well due to sufficient interfacial joint strength. Moreover, there was little structure damage or accumulating thermal stresses because of the natural properties of compressive seals during thermal cycles. Figure 6c,d show EDS profiles between interconnect, seals and cells. Mg and Si in seals were seldom observed in interconnect and cell. Similarly, Ni, Zr in cells and Fe, Cr in interconnect were almost not observed in seals, showing that elemental diffusions between seals and components were limited, which implies good compatibility. Therefore, the vermiculite shows good promise for application in stacks undergoing many thermal cycles and a wide input of gas pressure ranges. 

### 2.3. Estimation of Vermiculite Seals for Stack Application

**Figure 7:**
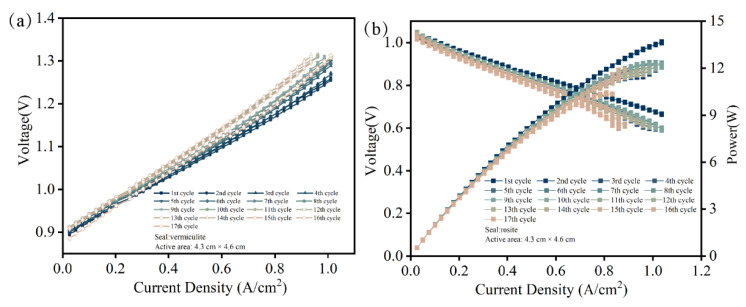
I–V curves in SOFC (**a**) and I-P-V curves in SOFC (**b**) of 1-cell during thermal cycles.

**Figure 8:**
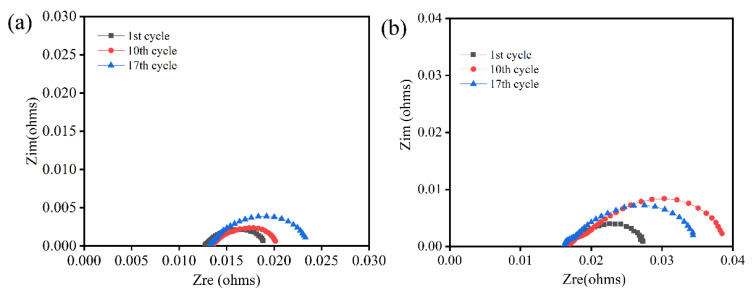
EIS of single cell stack at 750 °C at SOEC mode (**a**) and SOFC mode (**b**) under various.

The thermal stability of the stack highly depends on sealing performance. Using anode support cells and vermiculite seals, a single cell stack was assembled, and its performance was estimated. Figure 7a shows the current density–voltage (I–V) curves of one single stack in SOEC mode at 750 °C undergoing 17 thermal cycles. Obviously, the current density increased with elevating voltage. After ten thermal cycles, the maximum current density was 1.01 A/cm^2^ at the voltage of 1.3 V. Moreover, the performance of the stack stayed roughly stable during thermal cycles. Figure 7b shows the current density–voltage-power (I-P-V) curves of one single stack in SOFC mode at 750 °C and indicates a peak power of 14.19 W under a current density of 1.03 A/cm^2^. The power of the stack slightly decreased during thermal cycles, mainly due to a poor interfacial contact between the cell cathode and interconnect. Furthermore, the open circuit voltage (OCV) remained at around 1.04 V during 17 thermal cycles, which is close to Nernst potentials. It was obvious that the seals exhibited good sealing performance. The stack performance confirmed that the vermiculite seals can meet the structural support and sealing requirements. In order to verify the reversibility of one single cell stack, the EIS were measured under OCV conditions. As presented in Figure 8, the values of ohmic resistance stayed stable during thermal cycles. However, the polarization resistance slightly increased after various thermal cycles, which corresponded with cell performance. Combined with leakage rates and electrochemical performance, the vermiculite seals were indicated to be a suitable application during the thermal cycle in the RSOC stack.

## 3. Materials and Methods

Vermiculite is a phyllosilicate mineral which is commercially supplied by Zhejiang CPS Cathay Packing & Sealing Co., Ltd. from Hangzhou in China. The obtained materials were divided into 0.50 mm, 0.70 mm, and 1.00 mm thicknesses, respectively. The relationship between thickness and compressive loads was analyzed for obtaining a good collocation degree in RSOC stacks. The experiment was conducted as follows: the seals were held at various compressive loads from 0.05 MPa to 0.5 MPa for ten minutes, and the thickness was then measured by micrometer and recorded. Then, the thermal properties of the seals were measured to obtain a reasonable heat treatment process during operation in stacks using differential thermal analysis (STA449F3, Netzsch, Germany) at a heating rate of 10 °C /min at temperatures ranging from 40 °C to 1000 °C. 

The leakage rates of seals under various compressive loads from 0.1 MPa to 0.3 MPa were measured at temperatures from 700 °C to 800 °C using self-developed equipment. The seals were cut into a window frame with an outer size of 7 cm × 7 cm and an inner size of 5 cm × 5 cm. The principle of leakage-rate testing was as follows. The N_2_ was selected as a medium that can accurately provide an evaluation of leakage rates. The gas was divided into two pathways, one of which provided a compressive load ranging from 0.1 MPa to 0.3 MPa, while the other path was for leakage rate measurement. The seals were placed into polished alloy to construct a chamber under various compressive loads. The testing gas was admitted into the chamber under input gas pressures ranging from 1 psi to 3 psi. When the leakage occurred at the seals, the flow meter displayed the gas mass flow. Once the gas flow reached equilibrium, the leakage rates were recorded by mass flow (Alicat KM9901 America, Alicat Scientific, Tucson, AZ, USA) with a precision of 0.0001 sccm [26]. The leakage rates under thermal cycles and long-term operation were measured. In order to evaluate the performance, the one cell stack using vermiculite was assembled. Seals with a length of 4.9 cm and width of 4.7 cm were prepared and placed between cells and the fixture. The cells were obtained from Elcogen, with an active area of 4.3 cm × 4.6 cm. The test was conducted at 750 °C under a compressive load of 0.3 MPa using H_2_ as fuel and air as oxidant. Then the power output, open circuit voltage and Electrochemical Impedance Spectroscopy (EIS) were obtained after every thermal cycle. Furthermore, the testing configurations were disassembled to identify possible leaking holes corresponding with analyzing leakage rates and cell performance. 

The microstructures of seals under various compressive loads were examined to ascertain the reason for the leakage using a LEO 1530vp scanning electron microscope (SEM). Accordingly, in order to analyze interfacial compatibility between the seals and adjacent components, the sandwiched structure samples were assembled to examine the cross-section microstructure. Sandwich structure specimens of metallic interconnect (S441 alloy)/seal/anode (NiO+YSZ) with dimensions of 20 × 10 mm were prepared and heat-treated between 200 °C and 750 °C for twenty thermal cycles under a compressive load of 0.3 MPa to simulate seal interfaces in the stack. The samples after twenty thermal cycles were mounted in Buhler epoxide and polished using a Buhler automatic polisher. The cleaned samples were characterized by SEM attached with an energy dissipation spectrum (EDS), to display interfacial structure and composition.

## 4. Conclusions

The effect of thickness and compressive loads on leakage rates of seals were investigated in RSOC stacks. The sealing performance of vermiculite seals in stacks were conducted during thermal cycles and long-term operation. Based on the obtained results, the following conclusions can be drawn:

(1) The sealing performance of vermiculite gradually deteriorated with increasing thickness and input gas pressure. The thinner seals had good sealing performance. The coordination between assembly and compression could achieve electrical contact between cells and interconnects as well as achieve sealing performance. Moreover, the higher the compressive load, the smaller the leakage rates under the same input gas pressure. The compressive load was carried out at seals, the possible holes were squeezed, and finally the leakage rates were lower. The temperature had less influence on sealing performance. Finally, thinner seals with a suitable compressive load exhibited a good sealing performance.
(2) To further examine the long-term stability of vermiculite, the seals underwent long-term operation at 750 °C, and twenty thermal cycles from 750 °C to 200 °C were conducted. With a fixed input gas pressure of 1 psi, 2 psi, and 3 psi, the leakage rates remained at around 0.009 sccm/cm, 0.017 sccm/cm and 0.028 sccm/cm during twenty thermal cycles. Additionally, the leakage rates remained at around 0.011 sccm/cm during 240 h. Simultaneously, elemental diffusions between seals and components were limited, implying good compatibility.
(3) The maximum current density was 1.01 A/cm^2^ at a voltage of 1.3 V after ten thermal cycles. Furthermore, the open circuit voltage (OCV) remained at around 1.04 V during 17 thermal cycles, which is close to Nernst potentials. Moreover, the performance of the stack stayed roughly stable during thermal cycles. The stack performance confirmed that the vermiculite seals can meet the structural support and sealing requirements. Therefore, the vermiculite shows good promise for application in stacks undergoing many thermal cycles and wide input gas pressure ranges.

## Data Availability

The data presented in this paper are available on request from the corresponding author.

## Sample Availability

Samples of the compounds are available from the authors.
